# Regional Assessment of Lower limb Amputations in sub-Saharan Africa (RAMPs): a prospective cohort study protocol

**DOI:** 10.1136/bmjopen-2025-107789

**Published:** 2025-10-02

**Authors:** Nick Moody, Becky Sandford, David C Bosanquet, Kathryn Chu, Rahel Assefa, Jack Hall, Tabiri Stephen, M Popplewell, Nebyou Seyoum, Justine Davies

**Affiliations:** 1 University of Birmingham, Birmingham, UK; 2 Guy’s and St Thomas’ NHS Trust, London, UK; 3 South East Wales Vascular Network, University Hospital of Wales, Cardiff, Wales; 4 School of Medicine, Cardiff University, Cardiff, UK; 5 Centre for Global Surgery, Stellenbosch University Faculty of Medicine and Health Sciences, Cape Town, South Africa; 6 Department of Surgery, St. Peter's Specialized Hospital, Addis Ababa, Ethiopia; 7 University for Development Studies, Tamale, Ghana; 8 Addis Ababa University, Addis Ababa, Ethiopia; 9 Department of Applied Health Sciences, College of Medicine and Health, University of Birmingham, Birmingham, UK

**Keywords:** Amputation, Surgical, Vascular surgery, Diabetic foot, Trauma, Chronic Limb-Threatening Ischemia

## Abstract

**Abstract:**

**Introduction:**

Major lower limb amputation, defined as an amputation above the level of the ankle joint, is a substantial cause of morbidity and mortality. Limited data exist on the burden, aetiology and outcomes of major lower limb amputations in sub-Saharan Africa (SSA). This is despite increasing rates of diabetes, peripheral arterial disease and trauma, with further projected increases in these conditions, which often precede major lower limb amputation. The Regional Assessment of Amputations in sub-Saharan Africa (RAMPs) study aims to address this knowledge gap by performing a multicentre, prospective study of major lower limb amputations across the region.

**Methods:**

We describe a prospective, multicentre observational cohort study enrolling patients undergoing major lower limb amputation at hospitals in SSA over a consecutive 6-month period. Consecutive patients will be included, and data will be collected from medical records until discharge, death or 30 days postoperatively, whichever is sooner. The primary outcome is in-hospital or 30-day mortality. Secondary outcomes include the aetiology of amputations and in-hospital complications. We will also examine systems and processes using a facility survey of each participating centre. The study will collect system-level, patient-level and outcome-level data. Our sample size calculation suggests 904 patients need to be recruited.

**Ethics and dissemination:**

The RAMPs study will provide a snapshot of the current outcomes and aetiology of major lower limb amputation in SSA. It will show if variation in outcomes and aetiology in patients in the region exists and provide information on the healthcare processes and systems in those who may be at risk of lower limb amputation. Ethical approval has been granted by the University of Birmingham (Science, Technology, Engineering and Mathematics Committee reference: ERN_2929-Jan2025) and the College of Surgeons of East, Central and Southern Africa (COSECSA Institutional review board reference COSECSA/REC/2025/07). Findings will be disseminated throughout the region at local, national and international conferences and through at least one peer-reviewed manuscript.

**STRENGTHS AND LIMITATIONS OF THIS STUDY**

- The Regional Assessment of Amputations in sub-Saharan Africa study will be an international collaborative study throughout sub-Saharan Africa.
- It will collect data from any hospital that performs major lower limb amputations in the region.
- Data on the type of amputation, aetiology and postoperative outcomes will be collected.
- This study will not analyse new treatment or management options as it is an observational study only.

## Background

 Major lower limb amputation, defined as an amputation above the level of the ankle joint, is a devastating outcome for individuals, families and society. This is true even in high-income countries, with amputation often resulting in an inability to work, poor long-term health outcomes and stigmatisation.^1^,^3^ In sub-Saharan Africa (SSA), where all countries are categorised as low- or middle-income countries (LMICs) according to the Organisation for Economic Co-operation and Development, the impact is likely to be more pronounced.^4^ Compared with high-income countries, health resources are lower, while infrastructure is scarce, leading to little support for those who are at risk of, and those who undergo, major lower limb amputation.^5^

Little evidence exists to define the extent to which major lower limb amputation contributes to morbidity and mortality in SSA, or even which conditions contribute to lower limb amputation in this region.^4 6 7^ Studies which have assessed the incidence, causes and outcomes of amputations in the region have been predominantly single centre and retrospective in nature.^7^,^10^

Whilst trauma is recognised as a significant cause of amputations in the region, diabetes and peripheral vascular disease incidence (both of which predispose to amputation) are rising significantly. The number of people with diabetes in SSA is projected to rise by 129% between 2021 and 2045, resulting in an estimated 55 million people living with diabetes.^11 12^ This rise in incidence and prevalence is unlikely to be matched by diagnostic and treatment strategies, with a rising burden of end-stage complications, including limb-related pathology, for which amputation is the endpoint.^11 12^ It is estimated that at current baseline, 1161 disability-adjusted life years are lost per 1000 population over 10 years as a result of cardiovascular disease in diabetics in LMICs. ^13^

Amputation is often a marker of the quality of disease management, whilst also carrying a significant morbidity and mortality burden in its own right, with the WHO global diabetes compact suggesting lower-extremity amputation should be a target metric in the journey towards equitable and comprehensive care for those with diabetes in LMICs. 14,16

The Regional Assessment of Amputations in sub-Saharan Africa (RAMPs) study aims to address the gaps in knowledge in major lower limb amputations in SSA. The study is designed to enable understanding of basic demographics, indications, complications and in-hospital outcomes for people who have had an amputation. In addition, the amputation services provided by each hospital will be contextualised using a facility survey in each centre.

Findings from this study will allow comparison to well-documented outcomes in resource-rich settings and provide data for healthcare system planning and global advocacy.

### Primary objective

- To describe in-hospital, or up to 30 days, mortality outcomes of persons who undergo major lower limb amputations in SSA.

### Secondary objective

- To describe the aetiology of major lower limb amputations in countries in SSA.
- To describe the in-hospital, or up to 30-day, complications of those undergoing major lower limb amputations in SSA.

### Exploratory objective

- To understand the readiness of facilities to deliver quality limb salvage services.

## Methods

### Study design

This is a prospective multicentre observational cohort study of patients undergoing a major lower limb amputation. Major lower limb amputation will be defined as any amputation which is at or proximal to the ankle joint, including through ankle, below knee, through knee and above knee amputations, as well as hip disarticulation. We will recruit hospitals through pre-existing networks and use snowball sampling, as well as advertising on social media. Eligible consecutive patients within each hospital will be enrolled over a 6-month period and will be followed until discharge, death or 30 days postoperatively, whichever comes first. Hospitals will be able to enrol in the study and recruit patients for 6 months during a window between September 2025 and August 2026. Data on the number of health facilities previously visited by the participant, as well as the date of injury in those who have traumatic injuries, will be collected to understand the timeliness of care delivery.

Any hospital in SSA (as defined by the World Bank, online supplemental appendix 1) will be eligible to take part if they perform on average a minimum of one major lower limb amputation per month, regardless of indication. An additional hospital-level survey will be completed at all hospitals which enrol patients into the study. This will provide context for interpreting patient-level outcomes across different settings. The study will be overseen and managed by the study’s central committee.

### Participants

All patients who are admitted to hospital and undergo major lower limb amputation during the 6-month data collection period will be included. There are no age restrictions, although participating sites which manage only adults or children, or have age restrictions for recruitment to the studies will prespecify the age range of participants who will be recruited. Inclusion will be at the patient rather than the procedure level, allowing each patient to be included once in the data collection. A staged amputation (ie, a guillotine amputation followed by a formal closure) will be recorded as such. Patients who have an index amputation followed by a further amputation of the ipsilateral limb will have the second amputation recorded as a complication; if this was not to be a planned staged amputation (guillotine), not a ‘new’ data entry record. Contralateral limb amputation will also be recorded as a postoperative event rather than a new episode (this is anticipated to be a rare event).

### Outcome measures

The primary outcome is all-cause in-hospital mortality or 30 days for those who remain hospitalised 30 days after amputation.

Secondary outcomes include aetiology of amputation and complications occurring in-hospital or within 30 days of amputation. Complications include:

- In-hospital or 30-day return to theatre.
- Stump-related complication (wound dehiscence, infection or haematoma).
- Systemic complication (sepsis, stroke, myocardial infarction, pneumonia or contralateral limb amputation).
- Length of hospital stay or time from amputation to death in those who do not survive.

Aetiology will also be captured in data fields and will include traumatic injury (including the mechanism of injury and date of injury), diabetic foot infection, non-diabetic foot infection, peripheral vascular disease and malignancy and iatrogenic complications. Data on comorbidities, previous major and minor amputations, as well as previous health facility visits will also be captured.

### Data collection

Data will be collected on all patients who undergo a major lower limb amputation within the study period. Included data fields are based on work by similar studies and have been refined through iterative consultation with clinicians and researchers involved in amputation surgery across the region. Data will be collected from patient records at each centre. The patient will not be directly contacted during their inpatient stay or after the study unless contact is required to obtain consent for data collection. Data will be collected directly onto REDCap cloud, a well-established secure web-based system, or recorded temporarily on a hard copy data collection form prior to upload onto REDCap, as determined at each centre. Variables have been selected for pragmatic reasons; to address important questions whilst minimising the burden of data collection and the risk of participant identification.

All data entered into the REDCap system is anonymised. Access to anonymised data for analysis will be limited to named researchers (study authors) working on the study. Collaborators at each centre will have access to their own institutional data.

All collaborators (local leads, local collaborators and data collectors (up to five per facility)) will be listed individually as PubMed-citable collaborator status authors under the collaborator authorship name as outlined in the Guidelines for Standardising Reporting of Authorship in Collaborative Research.^17^

### Statistical analysis

A formal statistical analysis plan will be produced prior to complete data capture and database lock. However, in brief, data will be analysed using Stata/SE V.18.5. The mean and SD will be reported for all normally distributed variables. The median and IQR will be reported for non-normally distributed variables. Binary and categorical variables will be summarised as numbers and proportions. Primary and secondary outcomes will be described for the total sample, and disaggregated by age, sex, country income level (low-income, lower-middle income, upper-middle income, as defined by the World Bank at the time of study), type of amputation (hindquarter amputation, above-knee amputation, through-knee amputation, below-knee amputation and through-ankle amputation), indication for amputation and hospital types (district hospital, regional hospital, tertiary referral hospital).

Analysis to assess factors associated with the primary and secondary outcomes will be conducted using multivariable logistic modelling. Unadjusted crude estimates will be reported. Data will be adjusted for confounding factors, including age, sex and American Society of Anaesthesiologists score. Potential effect modifiers will also be adjusted for, including the method of anaesthesia.

As there is a lack of data on outcomes after major lower limb amputation in SSA, a sample size estimation is based on observational data from other LMICs. A review of the literature ascertained a 30-day mortality rate of approximately 15% after major lower limb amputation in LMICs. To detect this mortality rate with a 95% CI no wider than 5 percentage points, a sample size of 822 is required. Considering a 10% loss of data due to quality, we will aim for a sample size of 904. ^18^

From local experience, it is estimated that an average of one amputation a month will be performed in secondary level hospitals and five per month in tertiary hospitals. If 60 hospitals are included (30 tertiary and 30 secondary centres), capturing 180 patients per month, data collection will continue at each centre for 6 months to capture 1080 patients.

Data limits and data flow structures will ensure erroneous data entry is minimised. Data quality checks, based on cases entered and case completeness, will be carried out and data collection teams will be informed of their data quality each week. Patient-level data with more than 30% missing data points will be excluded in a sensitivity analysis. This is because missing values are expected in such a study, particularly given its focus on LMICs. Exclusion of centres which are more affected by data missingness may introduce bias. Multiple imputation methods (multiple imputation by chained equations or multiple imputation with denoising autoencoders) will be considered for the remaining data.

### Patient and public involvement

The research priorities set out in this study reflect those that have been determined by the James Lind Alliance priority setting partnership, a validated approach to developing key research questions.^19^

### Ethical and dissemination

Overarching ethical approval for the study, including the consent procedure, has been provided by the University of Birmingham ethical review board (Science, Technology, Engineering and Mathematics Committee review board review reference ERN_2929-Jan2025). Further ethical approval has been granted by the College of East, Central and Southern Africa institutional review board (reference COSECSA/REC/2025/07). This covers a large number of affiliated institutions throughout the region. Depending on local regulation, this study can be registered as a service evaluation or as research. Further local ethics committee and/or institutional review board approvals may be required, in which case, these will be applied for and in place prior to data collection period. As this study can be registered as an audit or service evaluation, informed consent may not be required, although this depends on local protocols. If local protocols or regulations require it, informed consent will be obtained from participants prior to enrolment in the study.

The study will be reported following the Strengthening the Reporting of Observational Studies in Epidemiology statement guidelines and checklists.^20^

Results will be presented at academic and clinical conferences in East, West and Southern Africa (eg, College of Surgeons of East, Central and Southern Africa Annual Conference, West African College of Surgeons Annual Conference). This study will result in at least one peer-reviewed publication.

## Supplementary material

10.1136/bmjopen-2025-107789 online supplemental file 1
