# Supplementary material for: Regional Assessment of Lower limb Amputations in sub-Saharan Africa (RAMPs): a prospective cohort study protocol
Source: BMJ Open. 2025 Oct 2;15(10):e107789. doi: 10.1136/bmjopen-2025-107789 (PMC12496064; doi:10.1136/bmjopen-2025-107789)
Supplement: online supplemental file 1 [file bmjopen-15-10-s001.docx]

# **R**egional **A**ssessment of Lower limb **Amp**utations in sub-**S**aharan Africa (RAMPs); a Prospective Cohort Study Protocol

Appendix 1.

List of countries in sub-Saharan Africa as defined by World Bank

- (Angola, Benin, Botswana, Burkina Faso, Burundi, Cabo Verde, Cameroon, Central African Republic, Chad, Comoros, Congo, Democratic Republic of, Congo, Republic of, Cote d'Ivoire, Equatorial Guinea, Eritrea, Eswatini (Formerly Known as Swaziland), Ethiopia, Gabon, Gambia, The, Ghana, Guinea, Guinea-Bissau, Kenya, Lesotho, Liberia, Madagascar, Malawi, Mali, Mauritania, Mauritius, Mozambique, Namibia, Niger, Nigeria, Rwanda, Sao Tome and Principe, Senegal, Seychelles, Sierra Leone, Somalia, South Africa, South Sudan, Sudan, Tanzania, Togo, Uganda, Zambia, Zimbabwe)
