# Supplementary material for: Regional Assessment of Lower limb Amputations in sub-Saharan Africa (RAMPs): a prospective cohort study protocol
Source: BMJ Open. 2025 Oct 2;15(10):e107789. doi: 10.1136/bmjopen-2025-107789 (PMC12496064; doi:10.1136/bmjopen-2025-107789)
Supplement: Supplementary file 2 [file bmjopen-2025-107789.reviewer_comments.pdf]

PEER REVIEW HISTORY

BMJ Open publishes all reviews undertaken for accepted manuscripts. Reviewers are asked to complete a checklist review form and are provided with free text boxes to elaborate on their assessment. These free text comments are reproduced below.

ARTICLE DETAILS

Title (Provisional)

Regional Assessment of Lower limb Amputations in sub-Saharan Africa (RAMPs); a Prospective Cohort Study Protocol

Authors

Moody, Nick; Sandford, Becky; Bosanquet, David C; Chu, Kathryn; Assefa, Rahel; Hall, Jack; Stephen, Tabiri; Popplewell, M; Nebyou, Abebe; Davies, Justine

---

VERSION 1 - REVIEW

**Reviewer**	**1**
**Name**	**de Mestral, Charles**
**Affiliation**	**St Michael's Hospital, Surgery**
**Date**	**24-Aug-2025**
**COI**	**None**

I commend the authors for working to generate new data on this important public health topic and source of morbidity.

My main comment is that the cover letters and protocol mention: " there is scant evidence on who gets amputations" and that the results will have value for amputation prevention. One of the 2ry objectives also focuses on " aetiology of major lower limb amputations in countries". Yet the methods only describe the post-op outcomes and not how the aetiology will be captured/coded.

1) The methods should describe how the aetiology will be captured/coded.

2) It would also be useful if able to describe past health care encounters that may reflect opportunity or efforts for limb salvage (has a person with diabetes had multiple encounters where a foot complication might have been prevented?, are most amputation a result of a recent injury/wound without any effort to salvage the limb?)

VERSION 1 - AUTHOR RESPONSE

Reviewer: 1
Dr. Charles de Mestral, St Michael's Hospital

Comments to the Author:
I commend the authors for working to generate new data on this important public health topic and source of morbidity.

My main comment is that the cover letters and protocol mention: " there is scant evidence on who gets amputations" and that the results will have value for amputation prevention. One of the 2ry objectives also focuses on " aetiology of major lower limb amputations in countries". Yet the methods only describe the post-op outcomes and not how the aetiology will be captured/coded.

1) The methods should describe how the aetiology will be captured/coded.

Thank you for highlighting this omission. We have added that we will be capturing aeitology in our data collection:
Aetiology will also be captured in data fields and will include traumatic injury (including the mechanism of injury), Diabetic foot infection, non-diabetic foot infection, peripheral vascular disease and malignancy.

2) It would also be useful if able to describe past health care encounters that may reflect opportunity or efforts for limb salvage (has a person with diabetes had multiple encounters where a foot complication might have been prevented?, are most amputation a result of a recent injury/wound without any effort to salvage the limb?)

Thank you for this comment, it is indeed interesting to try and understand whether missed opportunities have contributed to amputation. Our data collection fields do also include a section which, in the case of traumatic injury, asks for the date of injury to attempt to quantify the gap between injury date, presentation to hospital, and amputation. We have also included a question in our data collection on the number of prior health facilities visited.

We are planning a further in-depth follow-up study which will examine the delays in seeking healthcare for those who undergo amputation in Zambia. We were keen not to overburden data collectors in this study and therefore excluded detailed questions on previous health encounters. We have added the following to the manuscript in the methods section to highlight this:

Data on the number of health facilities previously visited by the participant, as well as date of injury in those who have traumatic injuries will be collected to understand the timeliness of care delivery.

VERSION 2 - REVIEW

**Reviewer**	**1**
**Name**	**de Mestral, Charles**
**Affiliation**	**St Michael's Hospital, Surgery**
**Date**	**08-Sep-2025**

COI

Thank you for the responses.

re: "Aetiology will also be captured in data fields and will include traumatic injury

(including the mechanism of injury), Diabetic foot infection, non-diabetic foot

infection, peripheral vascular disease and malignancy. "

I appreciate not overburdening the data collectors but would suggest a more detailed plan for data capture around potential etiologies contributing to amputation. Need to consider/capture:

1) potential overlap of etiologies (diabetes and PAD; trauma and diabetes)

2) broader range of etiologies (iatrogenic complication of foot/ankle surgery, deep vein thrombus/venous ulceration, cancer)

3) simple understanding of time from the initial insult (wound, trauma, etc.) and the amputation. e.g. within last 3 months or more chronic

---

VERSION 2 - AUTHOR RESPONSE

Reviewer: 1

Dr. Charles de Mestral, St Michael's Hospital

Comments to the Author:

Thank you for the responses.

re: "Aetiology will also be captured in data fields and will include traumatic injury

(including the mechanism of injury), Diabetic foot infection, non-diabetic foot

infection, peripheral vascular disease and malignancy. "

I appreciate not overburdening the data collectors but would suggest a more detailed plan for data capture around potential etiologies contributing to amputation. Need to consider/capture:

1) potential overlap of etiologies (diabetes and PAD; trauma and diabetes)

We anticipate that most amputations will have a primary indication, and whilst there will be overlapping aetiologies in some cases, we hope we will be able to capture this by capturing co-morbidities, which are also being collected as a

variable. For instance, we will capture those with diabetes who have a traumatic amputation. We have made this clear by altering the text on page 7 (see answer to point 3)

2) broader range of etiologies (iatrogenic complication of foot/ankle surgery, deep vein thrombus/venous ulceration, cancer)

We will indeed be capturing all of the above aetiologies, as well as including a 'other…free text section' to ensure that we do not exclude aetiologies which we may have failed to consider.

3) simple understanding of time from the initial insult (wound, trauma, etc.) and the amputation. e.g. within last 3 months or more chronic

Thank you for these comments. This is indeed also included in our data collection tool, which has a prompt for date of injury in those who undergo a traumatic amputation, as this is something which is likely to be routinely collected by clinicians when assessing a patient. We have again made this clear in the manuscript by modifying a paragraph on page 7 to read as follows:

Aetiology will also be captured in data fields and will include traumatic injury (including the mechanism of injury and date of injury), Diabetic foot infection, non-diabetic foot infection, peripheral vascular disease and malignancy and iatrogenic complications. Data on co-morbidities, previous major and minor amputations, as well as previous health facility visits will also be captured.
